# Cadherin–catenin expression in primary colorectal cancer: a survival analysis

**DOI:** 10.1038/sj.bjc.6690461

**Published:** 1999-06

**Authors:** T J Hugh, S A Dillon, B A Taylor, M Pignatelli, G J Poston, A R Kinsella

**Affiliations:** Cellular Oncology Group, Department of Surgery, University of Liverpool, Liverpool L69 3GA, UK; Department of Pathology, Royal Post-Graduate Medical School, Hammersmith Hospital, Du Cane Road, London W12 0HS, UK

**Keywords:** cadherin–catenin complex, colorectal cancer, β-catenin, nuclear expression

## Abstract

Both cell adhesion and cell signalling events are mediated by components of the cadherin–catenin complex. Loss of expression of the components of this complex have been shown to correlate with invasive behaviour in many tumour types although their exact role in colorectal cancer remains unclear. Immunohistochemical analysis of the expression of components of the cadherin–catenin complex in colorectal cancers from 60 patients was undertaken. Loss of memberanous expression of E-cadherin, α-catenin and β-catenin was demonstrated in 52%, 85% and 40% of tumours respectively. Focal nuclear expression of β-catenin (< 75% of cells per section), usually associated with cytoplasmic expression, was clearly demonstrated in 19 (32%) tumours while widespread nuclear expression (> 75% of tumour cells per section) was seen in 11 (18%) tumours. Loss of membranous α-catenin expression significantly correlated with tumour de-differentiation (*P* = 0.009). There was a trend towards an association between advanced tumour stage and loss of membranous expression of α-catenin or β-catenin, although these associations were not statistically significant. Univariate analysis revealed that advanced Dukes' stage, tumour de-differentiation, loss of membranous β-catenin expression, cytoplasmic β-catenin expression and widespread nuclear expression of β-catenin all correlated with short survival following apparently curative resection of the primary tumour. However, only Dukes' stage (*P* = 0.002), tumour grade (*P* = 0.02) and widespread nuclear expression of β-catenin (*P* = 0.002) were independent predictors of short survival. Disturbed growth signalling events in colorectal tumours are thought to result in nuclear accumulation of β-catenin. Consequently, tumours with widespread nuclear expression of β-catenin are likely to have severely abnormal growth characteristics, and which therefore might be predictive of short survival in these patients. © 1999 Cancer Research Campaign


*British Journal of Cancer* (1999) **80**(7), 1046–1051
© 1999 Cancer Research Campaign
Article no. bjoc.1998.0461

### Cadherin–catenin expression in primary colorectal cancer: a survival analysis

**TJ Hugh[1], SA Dillon[1], BA Taylor[1], M Pignatelli[2], GJ Poston[1] and AR Kinsella[1]**

[1]Cellular Oncology Group, Department of Surgery, University of Liverpool, Liverpool L69 3GA, UK; [2]Department of Pathology, Royal Post-Graduate Medical School, Hammersmith Hospital, Du Cane Road, London W12 0HS, UK

**Summary** Both cell adhesion and cell signalling events are mediated by components of the cadherin–catenin complex. Loss of expression of the components of this complex have been shown to correlate with invasive behaviour in many tumour types although their exact role in colorectal cancer remains unclear. Immunohistochemical analysis of the expression of components of the cadherin–catenin complex in colorectal cancers from 60 patients was undertaken. Loss of memberanous expression of E-cadherin, α-catenin and β-catenin was demonstrated in 52%, 85% and 40% of tumours respectively. Focal nuclear expression of β-catenin (< 75% of cells per section), usually associated with cytoplasmic expression, was clearly demonstrated in 19 (32%) tumours while widespread nuclear expression (> 75% of tumour cells per section) was seen in 11 (18%) tumours. Loss of membranous α-catenin expression significantly correlated with tumour de-differentiation ($P$ = 0.009). There was a trend towards an association between advanced tumour stage and loss of membranous expression of α-catenin or β-catenin, although these associations were not statistically significant. Univariate analysis revealed that advanced Dukes' stage, tumour de-differentiation, loss of membranous β-catenin expression, cytoplasmic β-catenin expression and widespread nuclear expression of β-catenin all correlated with short survival following apparently curative resection of the primary tumour. However, only Dukes' stage ($P$ = 0.002), tumour grade ($P$ = 0.02) and widespread nuclear expression of β-catenin ($P$ = 0.002) were independent predictors of short survival. Disturbed growth signalling events in colorectal tumours are thought to result in nuclear accumulation of β-catenin. Consequently, tumours with widespread nuclear expression of β-catenin are likely to have severely abnormal growth characteristics, and which therefore might be predictive of short survival in these patients.

**Keywords:** cadherin–catenin complex; colorectal cancer; β-catenin; nuclear expression

The cadherin–catenin cell adhesion complex mediates homotypic cell–cell adhesion and is known to play a fundamental role in embryonic development, morphogenesis and maintenance of the epithelial phenotype (Takeichi, 1995). Fluctuations in cell–cell adhesion are thought to be key events in the progression from localized malignancy to metastatic disease and several in-vitro studies have confirmed that E-cadherin acts as a tumour suppressor protein (Vleminckx et al, 1991; Kinsella et al, 1994). Reduced E-cadherin expression has been reported in a variety of human tumours and in many cases these changes correlate with advanced disease (Shiozaki et al, 1996). However, the evidence in relation to colorectal cancer is less convincing. Some colorectal tumours expressing E-cadherin metastasize and some liver metastases strongly express E-cadherin (Kinsella et al, 1993; Gagliardi et al, 1995). In tumours with normal expression of E-cadherin, perturbations of the cadherin cell adhesion system may be due to abnormal expression or function of the associated catenins (Takeichi, 1993).

Functional cadherin-dependent cell adhesion requires the formation of complexes between E-cadherin and the cytosolic proteins α-catenin, β-catenin and plakoglobin (γ-catenin) (Ozawa and Kemler, 1992). β-Catenin and plakoglobin share amino acid homology and bind, in a mutually exclusive fashion, to the cytoplasmic domain of E-cadherin (Jou et al, 1995). β-Catenin competes with plakoglobin for binding to α-catenin which, in turn, is linked to the actin cytoskeleton (Knudsen et al, 1995).

Recently it has become evident that there are cadherin-independent, as well as cadherin-bound, catenin pools and that these two pools may serve different functions (Hinck et al, 1994; Jou et al, 1995). In colorectal cancer cell lines, elevated cytoplasmic levels of β-catenin are down-regulated by the product of the tumour suppressor gene APC, which has been shown to function in concert with glycogen synthase kinase-3β (GSK-3β) to regulate levels of uncomplexed β-catenin (Rubinfeld et al, 1996). This regulatory mechanism is also known to be part of a signalling pathway for β-catenin which controls cell growth and proliferation in vertebrate embryos (Gumbiner, 1995). Despite numerous studies documenting expression of the individual components of the cadherin–catenin cell adhesion complex in various tumour types, the role of the complex in tumourigenesis is still not fully understood. In colorectal cancer, the possible dual role of β-catenin in cell adhesion and in growth signalling events, and the relationship of these functions to patient outcome, remains to be clarified.

The aim of this study was to examine the expression of the components of the cadherin–catenin cell adhesion complex in colorectal cancer and attempt to correlate these findings with standard clinico-pathological parameters and patient survival.

*Received 20 May 1998*
*Revised 8 September 1998*
*Accepted 7 October 1998*

*Correspondence to:* TJ Hugh, Department of Surgery, Wallace Freeborn Professorial Block, Royal North Shore Hospital, St Leonards, NSW 2065, Australia

**Table 1** Membranous expression of the cadherin–catenin complex ($n$ = 60)

| | Loss of membranous expression (%) | Strong membranous expression (%) |
|---|---|---|
| E-cadherin | 31 (52) | 29 (48) |
| α-catenin | 51 (85) | 9 (15) |
| β-catenin | 24 (40) | 36 (60) |

### MATERIALS AND METHODS

### Tumour specimens

Tumour samples were obtained from 60 patients who underwent apparently curative resection of a primary colorectal carcinoma at the Royal Liverpool University Hospital between 1989 and 1994. The present series includes 44 patients whose colorectal tumours were previously examined for E-cadherin expression (Kinsella et al, 1993). Only one patient in the series had familial adenomatous polyposis and no patient received chemotherapy or radiotherapy prior to undergoing resection of the primary tumour. Tumour specimens and samples of normal colonic epithelium were collected at the time of resection and were snap-frozen and stored in liquid nitrogen until required. Clinico-pathological information and survival data were obtained from the hospital records, from death certificates or by contact with the local family practitioner.

### Immunohistochemistry

The avidin–biotin indirect immunoperoxidase method was used for immunohistochemical staining, as described previously (Andrews et al, 1997). Briefly, frozen sections (6 μM) of tumour samples were cut and fixed in ice-cold acetone followed by treatment with 1.2% hydrogen peroxide in methanol for 10 min to block endogenous peroxidase activity. The sections were incubated with either anti-E-cadherin (HECD-1) monoclonal IgG (R & D Systems Europe, Abingdon, UK) diluted 1:200 in Tris-buffered saline pH 7.6 (TBS), or anti-β-catenin monoclonal IgG (Transduction Laboratories, Lexington, KY, USA) diluted 1:100 in TBS, or anti-α-catenin monoclonal IgG (Transduction Laboratories, Lexington, KY, USA) diluted 1:20 in TBS. The sections were then thoroughly washed with TBS followed by addition of a biotinylated anti-mouse IgG (Amersham Life Sciences, UK). After incubation with ABC reagent (Dako, UK) the signal was finally developed with diaminobenzidine containing 0.01% hydrogen peroxide. Normal colonic epithelium was used as an internal positive control; negative controls consisted of adjacent sections in which the primary antibody was replaced by either non-specific mouse IgG or TBS.

A

B

C

D

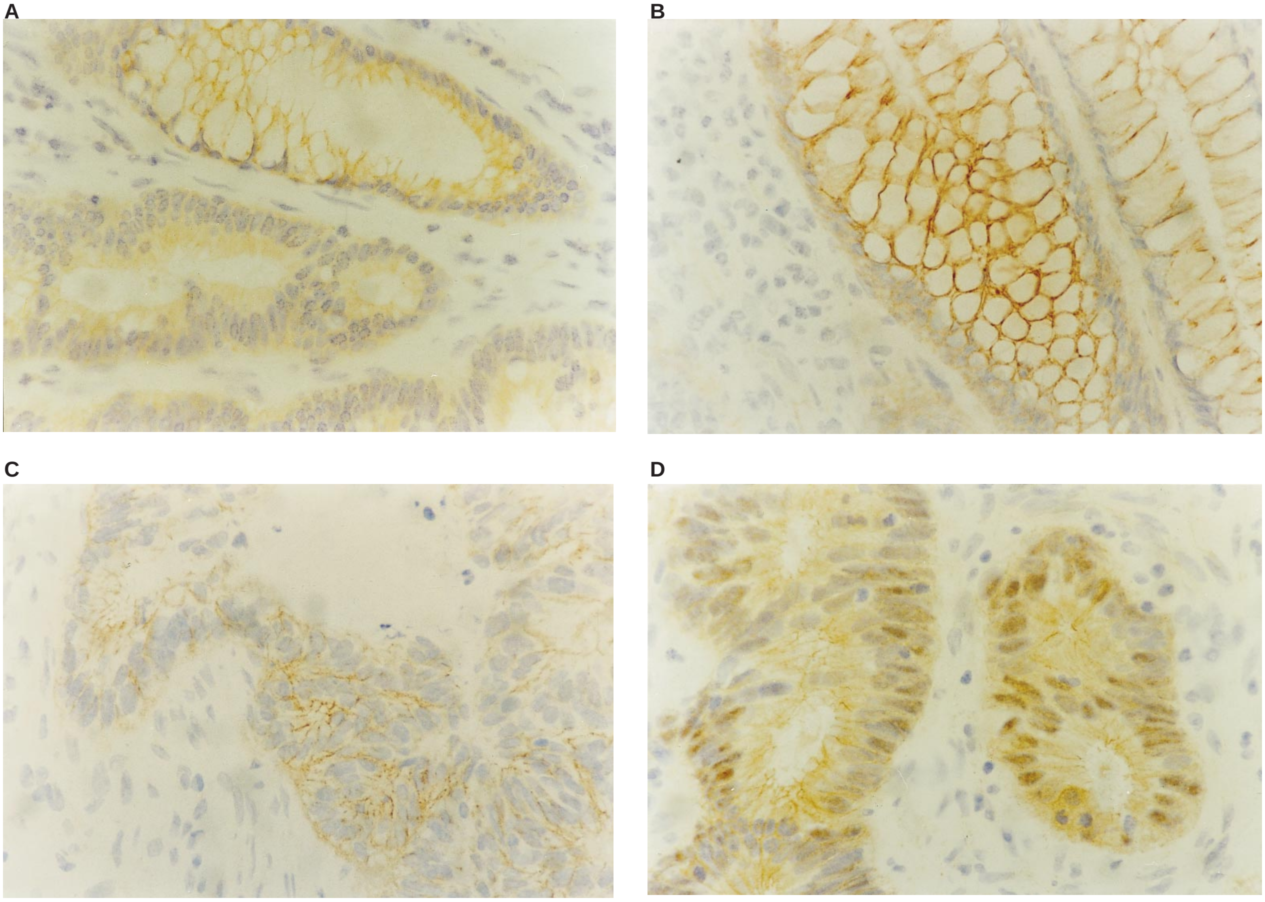

**Figure 1** (**A**) Cytoplasmic expression of α-catenin in a colonic carcinoma with normal colon internal control above (600×). (**B**) Strong membranous expression of β-catenin in normal colon (600×). (**C**) Weak membranous expression of β-catenin in a colonic carcinoma (600×). (**D**) Cytoplasmic, nuclear, and weak membranous expression of β-catenin in a colonic carcinoma (600×)

*© 1999 Cancer Research Campaign*

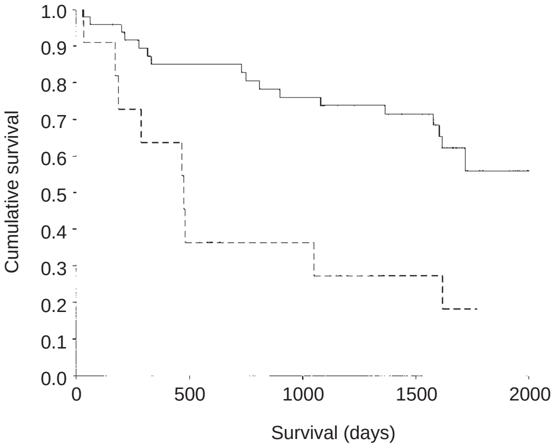

**Figure 2** Kaplan–Meier survival curve showing a statistically significant survival advantage in patients with tumours displaying focal or absent nuclear expression of β-catenin (*n* = 49, —) compared with those displaying widespread nuclear expression of β-catenin (*n* = 11, - - -) (log-rank test, $\chi^2_1$ = 9.74, *P* = 0.002)

**Table 2** Non-membranous expression of the cadherin–catenin complex (*n* = 60)

| ***n* = 60** | **Non-membranous distribution of the cadherin–catenin complex (%)** |
|---|---|
| E-cadherin | 5 (9) Faint cytoplasmic staining |
| α-catenin | 25 (42) Cytoplasmic staining |
| β-catenin | 45 (75) Diffuse cytoplasmic staining |
| | 19 (32) Focal nuclear staining (< 75% of the section) |
| | 11 (18) Widespread nuclear staining (> 75% of the section) |

### Scoring

Sections were examined under light microscopy and the predominant pattern of immunostaining within each section was used to determine the final score. Tumours displaying well-localized membranous staining similar to that seen in normal colorectal mucosa were scored as (2+); those displaying reduced membranous immunoreactivity were scored as (1+); tumours with no immunolabelling were scored as (0). Tumours were additionally subdivided into either cytoplasmic (+c) or nuclear (+n) localization. Nuclear expression of tumour cells was arbitrarily scored as either widespread (> 75% of cells per section), focal (< 75% of cells per section) or absent (no nuclear staining). All tumours were re-assessed for differentiation status by a single pathologist independent of the original pathology report.

### Statistical analysis

Kendall's Tau-b correlation was used to measure relationships between the expression of the components of the cadherin–catenin complex. The $\chi^2$ test, or Fisher's exact test where appropriate, was used to compare antigen expression with standard clinico-pathological parameters. Overall patient survival rates were calculated by the Kaplan–Meier method and the log-rank test was used for differences between survival curves. A *P*-value of < 0.05 was accepted as statistically significant. Variables were subjected to univariate analysis and after confirmation that the log–log plots did not deviate from the proportional hazards assumption, significant variables were subjected to multivariate analysis using the Cox regression model. The SPSS for Windows statistical package (Version 6.1.2, 2nd May, 1995) was used for all statistical analyses.

**Table 3** Relationship between loss of membranous expression of the cadherin–catenin complex (1 + or 0) and tumour grade

| **Tumour grade** | **Total** | **E-cadherin (%) 1 + or 0** | **α-catenin (%) 1 + or 0** | **β-catenin (%) 1+** |
|---|---|---|---|---|
| Well | 8 | 3 (38) | 4 (50) | 1 (13) |
| Moderate | 46 | 24 (52) | 41 (89) | 20 (43) |
| Poor | 6 | 4 (67) | 6 (100) | 3 (50) |
| *P*-value | | 0.55 | 0.009 | 0.22 |

**Table 4** Relationship between loss of membranous expression of the cadherin–catenin complex (1+ or 0) and Dukes' stage

| **Dukes' stage** | **Total** | **E-cadherin (%) 1+ or 0** | **α-catenin (%) 1+ or 0** | **β-catenin (%) 1+** |
|---|---|---|---|---|
| A | 6 | 4 (67) | 4 (67) | 0 (0) |
| B | 35 | 14 (40) | 28 (80) | 14 (40) |
| C | 19 | 13 (68) | 19 (100) | 10 (53) |
| *P*-value | | 0.10 | 0.06 | 0.07 |

### RESULTS

### Normal colorectal tissue

All normal mucosal specimens showed distinct, evenly distributed immunostaining for E-cadherin, α-catenin (Figure 1A) and β-catenin (Figure 1B) along the intercellular borders, but not at the cell surface facing the basement membrane. All three antigens displayed some basal cytoplasmic expression in colorectal cells located towards the luminal surface of the crypts. There was no detectable nuclear staining of either E-cadherin or the catenins in any section of normal colonic mucosa.

### Tumour tissue

Only six (10%) tumours displayed normal membranous expression (2+) of all three components of the cadherin–catenin complex, while five (8%) tumours displayed reduced or absent expression (1+ or 0) of all components of the complex. Tables 1 and 2 provide a summary of the immunohistochemical findings.

Loss of expression of E-cadherin was manifest as either discontinuous, weak immunolabelling at the intercellular borders or localized expression at the apicolateral cell junctions only. In some tumours faint cytoplasmic expression of E-cadherin was scattered throughout the cell. Overall, expression of α-catenin (Figure 1A) in tumours was less marked than either E-cadherin or β-catenin; in 25 (42%, 95% confidence interval (CI) 30–54) tumours there was complete absence of α-catenin expression. With regard to β-catenin, only 15 (25%, 95% CI 19–31) tumours displayed purely membranous expression, equivalent to that seen in normal colon; in the majority of tumours subcellular β-catenin staining was observed. Overall, there was good correlation between loss of membranous expression of β-catenin (Figure 1C) and widespread nuclear staining of β-catenin (Figure 1D) (Fisher's exact test,

© 1999 Cancer Research Campaign two-tailed, $P$ = 0.04). However, this was not an absolute finding and within some sections tumour cells displayed both nuclear and strong membranous expression of β-catenin.

There was no apparent statistical relationship between any form of β-catenin expression and either E-cadherin or α-catenin expression. However, there was a statistically significant relationship between E-cadherin and α-catenin expression (Kendall's Tau-b, R = 0.25, $P$ = 0.05), with tumours displaying loss of membranous E-cadherin expression often also displaying loss of α-catenin expression.

### Relationship with clinico-pathological parameters

Poorly differentiated tumours displayed greater loss of α-catenin expression when compared with moderately or well-differentiated tumours ($\chi^2_2$ = 9.4, $P$ = 0.009). Although there was a trend towards greater loss of membranous E-cadherin or β-catenin expression in less well-differentiated tumours, these findings were not statistically significant (Table 3). Tumours with reduced or absent membranous expression of α-catenin or β-catenin were more likely to develop regional lymph node metastases, although these associations did not reach statistical significance (Table 4). Furthermore, there was no apparent association between expression of the cadherin–catenin complex and patient gender or site of the primary tumour.

### Survival analysis

The median survival of the patients in this series from the time of primary tumour resection was 1720 days, with a 5-year actuarial survival of 46%. One patient died of an acute myocardial infarction immediately post-operatively (1.6% 30-day mortality) and two other patients died of non-cancer-related causes during subsequent follow-up. However, all three patients were known to be disease-free at the time of death. Two other patients who were lost to follow-up at 5 and 7 months post-operatively were also disease-free at the time of census.

As expected, both Dukes' staging and tumour grade were important prognostic factors. When subjected to univariate analysis, advanced Dukes' stage (log-rank test, $\chi^2_2$ = 19.75, $P$ = 0.0001) and tumour de-differentiation (log-rank test, $\chi^2_2$ = 10.03, $P$ = 0.007) correlated with short survival and death from metastatic colorectal cancer. In addition, loss of membranous expression of β-catenin (log-rank test, $\chi^2_1$ = 4.52, $P$ = 0.03), cytoplasmic expression of β-catenin (log-rank test, $\chi^2_1$ = 3.81, $P$ = 0.05), and widespread nuclear expression of β-catenin (log-rank test, $\chi^2_1$ = 9.74, $P$ = 0.0018, Figure 2) were also statistically associated with short survival and death from metastatic colorectal cancer. In contrast, there was no apparent association between long-term survival and either sex of the patient, site of the primary tumour, expression of E-cadherin or expression of α-catenin.

However, when the relevant significant variables were subjected to multivariate analysis only Dukes' staging ($P$ = 0.002), tumour grade ($P$ = 0.02) and widespread nuclear expression of β-catenin ($P$ = 0.002) were independent predictors of short survival. Patients with tumours displaying widespread nuclear staining of β-catenin (> 75% of tumour cells) had an overall 5-year survival of only 18% compared with 63% for patients whose tumours had focal or absent nuclear staining of β-catenin.

### DISCUSSION

In the present study, α-catenin expression in colorectal tumours was reduced or lost more frequently than either E-cadherin or β-catenin, and this reduction in expression correlated significantly with deteriorating tumour grade. Similar findings have been reported previously in several other tumour types (Kadowaki et al, 1994; Matsui et al, 1994; Takayama et al, 1994). The mechanisms resulting in loss of α-catenin expression in tumours are not fully understood. However, homozygous deletion of the α-catenin gene, resulting in loss of α-catenin expression, has been demonstrated in cell lines from both lung and prostatic carcinomas (Shimoyama et al, 1992; Morton et al, 1993). Furthermore, Yasui et al (1995) have reported complete deletion of the α-catenin gene in one of 10 gastric carcinomas examined. It has also been suggested that loss of α-catenin expression in tumours may relate to a post-transcriptional mechanism which is regulated by E-cadherin and which controls α-catenin protein expression (Nagafuchi et al, 1991). Dependence on E-cadherin for transcriptional regulation, as well as dependence on β-catenin for binding and incorporation into the cadherin–catenin complex may provide an explanation for the relatively early and frequent loss of α-catenin expression seen in many tumours. Consequently, loss of α-catenin might be the most sensitive indicator of cadherin–catenin complex dysfunction.

In contrast to previous reports, the present study has, for the first time, documented the relationship between expression of all three components of the cadherin–catenin complex in colorectal tumours and long-term survival. Others have reported an apparent association between loss of E-cadherin expression in colorectal tumours and advanced tumour stage and decreased survival (Dorudi et al, 1995); however, a similar relationship was not found in either an earlier study (Kinsella et al, 1993) or in the present one.

Loss of membranous β-catenin expression has been documented in a variety of tumour types, including colorectal cancer (Hashizume et al, 1996; Takayama et al, 1996; Hao et al, 1997*a*; Hiscox and Jiang, 1997; Jawhari et al, 1997). In our study, 40% of colorectal tumours displayed loss of membranous β-catenin expression and these changes correlated significantly with short survival of patients ($P$ = 0.033); a similar association has been previously reported in gastric and oesophageal adenocarcinomas (Jawhari et al, 1997; Krishnadath et al, 1997).

Nuclear expression of β-catenin in familial and sporadic colorectal tumours has recently been reported (Inomata et al, 1996; Takayama et al, 1996; Hao et al, 1997*b*; Valizadeh et al, 1997). Prior to this, subcellular localization of β-catenin was identified in mesodermal cells of vertebrate embryos during specific growth signalling events (Miller and Moon, 1996). Subsequently, Valizadeh and co-workers clearly demonstrated that early dysplastic colonic polyps, but not inflammatory or hyperplastic polyps, may display both cytoplasmic and nuclear localization of β-catenin (Valizadeh et al, 1997), suggesting that these changes are specific and early events in the development of colorectal cancer. The present study has extended these observations and demonstrates that up to 75% of sporadic colorectal cancers may display either cytoplasmic expression or nuclear localization of β-catenin. Furthermore, varying degrees of β-catenin nuclear expression are often found, with some tumours displaying focal staining only while others demonstrate more widespread staining.

Recent experimental and clinical evidence has demonstrated that cytoplasmic over-expression and nuclear translocation of β-catenin in colorectal tumour cells is usually a result of mutations in either the APC or β-catenin genes (Morin et al, 1997; Rubinfeld et al, 1997; Iwao et al, 1998). Furthermore, unregulated subcellular β-catenin protein has been shown to activate gene expression by complex formation with a family of nuclear transcription factors

*© 1999 Cancer Research Campaign*

(Behrens et al, 1996; Molenaar et al, 1996; Korinek et al, 1997). The finding of an association between widespread nuclear expression of β-catenin in tumour cells and short survival in patients with colorectal cancer (Figure 2) has not been reported previously. Unfortunately, in our study clinical information regarding sites of first recurrence of colorectal cancer was not available in all patients and therefore we cannot comment on whether widespread nuclear expression of β-catenin is more likely to be associated with either local, haematogenous or lymphatic metastases.

Failure to demonstrate loss of membranous expression of β-catenin as an independent predictor of survival conflicts with the findings of a recent study of patients with gastric cancer (Jawhari et al, 1997). Although the reasons for these differences are unclear, it is interesting to note from work done in our own laboratory (unpublished data) and by others (Jawhari et al, 1997), that nuclear expression of β-catenin is uncommon in gastric cancer compared with colorectal cancer, possibly because of less frequent APC mutations in gastric tumours.

In the present series of colorectal tumours, good correlation between loss of membranous β-catenin expression and widespread nuclear localization of β-catenin ($P$ = 0.04) was demonstrated. Similar reciprocity between membranous and nuclear staining of β-catenin has been demonstrated in colorectal adenomas and carcinomas recently (Hao et al, 1997*b*). However, in our study this relationship was not absolute and some tumours contained cells displaying both nuclear and strong membranous expression of β-catenin. Although there is evidence that the cadherin-binding and signalling activities of β-catenin may reside in separate regions of the protein (Gumbiner, 1995), the exact relationship between cadherin-bound β-catenin mediating cell adhesion and non-cadherin-bound β-catenin functioning as part of a growth signalling pathway remains unclear. It is possible the final tumour phenotype in colorectal cancer may depend on the relative functional activity of each of these regions.

In conclusion, this study demonstrates that loss of membranous expression of E-cadherin or β-catenin in colorectal tumours may not add prognostic information to standard clinico-pathological parameters. While α-catenin expression in colorectal cancers may reflect tumour differentiation status, our results suggest that it does not predict long-term patient outcome. In contrast, widespread nuclear expression of β-catenin in colorectal tumours, which possibly reflects unregulated growth signalling events, may distinguish tumours which behave aggressively. This preliminary report of a relationship between widespread nuclear expression of β-catenin and short survival in colorectal cancer needs to be confirmed in larger studies. It will be necessary to clarify the exact relationship between cell adhesion and cell signalling pools of β-catenin in colorectal cancer in order to understand the mechanisms underlying this finding.

### ACKNOWLEDGEMENTS

This work was funded by grants from the Royal Australasian College of Surgeons (TJH) and the North West Cancer Research Fund (ARK)

### REFERENCES

Andrews NA, Jones AS, Helliwell TR and Kinsella AR (1997) Expression of the E-cadherin–catenin cell adhesion complex in primary squamous cell cancer of the head and neck and their nodal metastases. *Br J Cancer* **75**: 1474–1480

Behrens J, von Kries JP, Kuhl M, Bruhn L, Wedlich D, Grosschedl R and Birchmeier W (1996) Functional interaction of β-catenin with the transcription factor LEF-1. *Nature* **382**: 638–642

Dorudi S, Hanby AM, Poulsom R, Northover J and Hart IR (1995) Level of expression of E-cadherin mRNA in colorectal cancer correlates with clinical outcome. *Br J Cancer* **71**: 614–616

Gagliardi G, Kandemir O, Liu D, Guida M, Benvestito S, Ruers TGM, Benjamin IS, Northover JMA, Stamp GWH, Talbot IC and Pignatelli M (1995) Changes in E-cadherin immunoreactivity in the adenoma–carcinoma sequence of the large bowel. *Virchows Arch* **426**: 149–154

Gumbiner BM (1995) Signal transduction by β-catenin. *Curr Opin Cell Biol* **7**: 634–640

Hao XP, Palazzo JP, Ilyas M, Tomlinson IPM and Talbot IC (1997*a*) Reduced expression of molecules of the cadherin/complex in the transition from colorectal adenoma to carcinoma. *Anticancer Res* **17**: 2241–2247

Hao XP, Tomlinson I, Ilyas M, Palazzo JP, Talbot IC (1997*b* ) Reciprocity between membranous and nuclear expression of beta-catenin in colorectal tumours. *Virchows Arch* **431**: 167–172

Hashizume R, Koizumi H, Ihara A, Ohta T and Uchikoshi T (1996) Expression of beta-catenin in normal tissue and breast carcinoma: a comparative study with epithelial cadherin and alpha-catenin. *Histopathology* **29**: 139–146

Hinck L, Nathke IS, Papkoff J and Nelson WJ (1994) Dynamics of cadherin/catenin complex formation – novel protein interactions and pathways of complex assembly. *J Cell Biol* **125**, 1327–1340

Hiscox S and Jiang WG (1997). Expression of E-cadherin, α, β, and γ-catenin in human colorectal cancer. *Anticancer Res* **17**: 1349–1354

Inomata M, Ochiai A, Akimoto S, Kitano S and Hirohashi S (1996) Alteration of β-catenin expression in colonic epithelial cells of familial adenomatous polyposis patients. *Cancer Res* **56**: 2213–2217

Iwao K, Nakamori S, Kameyama M, Imaoka S, Kinoshita M, Fukui T, Ishiguru S, Nakamura Y and Miyoshi Y (1998) Acivation of β-catenin gene by interstitial deletions involving exon 3 in primary colorectal carcinomas without adenomatous polyposis coli mutations. *Cancer Res* **58**: 1021–1026

Jawhari A, Jordan S, Poole S, Browne P, Pignatelli M and Farthing JG (1997) Abnormal immunoreactivity of the E-cadherin–catenin complex in gastric carcinoma: relationship with patient survival. *Gastroenterology* **112**: 46–54

Jou TS, Stewart DB, Stappert J, Nelson WJ and Marrs JA (1995) Genetic and biochemical dissection of protein linkages in the cadherin–catenin complex. *Proc Natl Acad Sci USA* **92**: 5067–5071

Kadowaki T, Shiozaki H, Inoue M, Tamura S, Oka H, Doki Y, Iihara K, Matsui S, Iwazawa T, Nagafuchi A, Tsukita S and Mori T (1994) E-cadherin and α-catenin expression in human esophageal cancer. *Cancer Res* **54**: 291–296

Kinsella AR, Green B, Lepts GC, Hill CL, Bowie G and Taylor BA (1993) The role of the cell–cell adhesion molecule E-cadherin in large bowel tumour cell invasion and metastasis. *Br J Cancer* **67**: 904–909

Kinsella AR, Lepts GC, Hill CL and Jones M (1994) Reduced E-cadherin expression correlates with increased invasiveness in colorectal carcinoma cell lines. *Clin Exp Metastatasis* **12**: 335–342

Knudsen KA, Soler AP, Johnson KR and Wheelock MJ (1995) Interaction of alpha-actinin with the cadherin–catenin cell–cell adhesion complex via α-catenin. *J Cell Biol* **130**: 67–77

Korinek V, Barker N, Morin PJ, Van Wichen D, de Weger R, Kinzler KW, Vogelstein B and Clevers H (1997) Constitutive transcriptional activation by a β-catenin–Tcf complex in APC−/− colon carcinoma. *Science* **275**: 1784–1787

Krishnadath KK, Tilanus HW, van Blankenstein M, Hop WCJ, Kremers ED, Dinjens WNM and Bosman FT (1997) Reduced expression of the cadherin–catenin complex in oesophageal adenocarcinoma correlates with poor prognosis *J Pathol* **182**: 331–338

Matsui S, Shiozaki H, Inoue M, Tamura S, Doki Y, Kadowaki T, Iwazawa T, Shimaya K, Nagafuchi A, Tsukita S and Mori T (1994) Immunohistochemical evaluation of α-catenin expression in human gastric cancer. *Virchows Arch* **424**: 375–381

Miller JR and Moon RT (1996) Signal transduction through β-catenin and specification of cell fate during embryogenesis. *Genes Dev* **10**: 2527–2539

Molenaar M, van de Wetering M, Oosterwegel M, Peterson-Maduro J, Godsave S, Korinek V, Roose J, Destree O and Clevers H (1996) XTcf-3 transcription factor mediates β-catenin-induced axis formation in Xenopus embryos. *Cell* **86**: 391–399

Morin PJ, Sparks AB, Korinek V, Barker N, Clevers H, Vogelstein B and Kinzler KW (1997) Activation of β-catenin-Tcf signalling in colon cancer by mutations in β-catenin or APC. *Science* **275**: 1787–1790.

Morton RA, Ewing CM, Nagafuchi A, Tsukita S and Isaacs WB (1993) Reduction of E-cadherin levels and deletion of the alpha-catenin gene in human prostate cancer cells. *Cancer Res* **53**: 3585–3590

 *© 1999 Cancer Research Campaign*

Nagafuchi A, Takeichi M and Tsukita S (1991) The 102 kd cadherin-associated protein: similarity to vinculin and post-transcriptional regulation of expression. *Cell* **65**: 849–857

Ozawa M and Kemler R (1992) Molecular organization of the uvomorulin–catenin complex. *J Cell Biol* **116**: 989–996.

Rubinfeld B, Albert I, Porfiri E, Fiol C, Munemitsu S and Polakis P (1996) Binding of GSK3β to the APC-β-catenin complex and regulation of complex assembly. *Science* **272**: 1023–1026

Rubinfeld B, Albert I, Porfiri E, Munemitsu S and Polakis P (1997) Loss of beta-catenin regulation by the APC tumor suppressor protein correlates with loss of structure due to common somatic mutations of the gene. *Cancer Res* **57**: 4624–4630

Shimoyama Y, Nagafuchi A, Fujita S, Gotoh M, Takeichi M, Tsukita S and Hirohashi S (1992) Cadherin dysfunction in a human cancer cell line – possible involvement of loss of alpha-catenin expression in reduced cell–cell adhesiveness. *Cancer Res* **52**: 5770–5774

Shiozaki H, Oka H, Inoue M, Tamura S and Monden M (1996) E-cadherin mediated adhesion system in cancer cells. *Cancer* **77**: 1605–1613

Takayama T, Shiozaki H, Inoue M, Tamura S, Oka H, Kadowaki T, Takatsuka Y, Nagafuchi A, Tsukita S and Mori T (1994) Expression of E-cadherin and α-catenin molecules in human breast cancer tissues and association with clinicopathological features. *Int J Oncol* **5**: 775–780

Takayama T, Shiozaki H, Shibamoto S, Oka H, Kimura Y, Tamura S, Inoue M, Monden T, Ito F and Monden M (1996) β-Catenin expression in human cancer. *Am J Pathol* **148**: 39–46

Takeichi M (1993) Cadherins in cancer: implications for invasion and metastasis. *Curr Opin Cell Biol* **5**: 806–811

Takeichi M (1995) Morphogenic roles of classic cadherins. *Curr Opin Cell Biol* **7**: 619–627

Valizadeh A, Karayiannakis AJ, El-Hariry I, Kmiot W and Pignatelli M (1997) Expression of E-cadherin-associated molecules (α-, β-, γ-catenin and p120) in colorectal polyps. *Am J Pathol* **150**: 1977–1984

Vleminckx K, Vakaet L Jr, Mareel M, Fiers W and van-Roy F (1991) Genetic manipulation of E-cadherin expression by epithelial tumour cells reveals an invasion suppressor role. *Cell* **66**: 107–119

Yasui W, Kuniyasu H, Akama Y, Kitahara K, Nagafuchi A, Ishihara S, Tsukita S and Tahara E (1995) Expression of E-cadherin, α-catenins and β-catenins in human gastric carcinomas – correlation with histology and tumour progression. *Oncol Rep* **2**: 111–117

*© 1999 Cancer Research Campaign* 
